# ECM-Dependent HIF Induction Directs Trophoblast Stem Cell Fate via LIMK1-Mediated Cytoskeletal Rearrangement

**DOI:** 10.1371/journal.pone.0056949

**Published:** 2013-02-21

**Authors:** Hwa J. Choi, Timothy A. Sanders, Kathryn V. Tormos, Kurosh Ameri, Justin D. Tsai, Angela M. Park, Julissa Gonzalez, Anthony M. Rajah, Xiaowei Liu, Diana M. Quinonez, Paolo F. Rinaudo, Emin Maltepe

**Affiliations:** 1 Department of Pediatrics, University of California San Francisco, San Francisco, California, United States of America; 2 Department of Biology, San Francisco State University, San Francisco, California, United States of America; 3 Department of Obstetrics, Gynecology and Reproductive Sciences, University of California San Francisco, San Francisco, California, United States of America; 4 Center for Reproductive Sciences, University of California San Francisco, San Francisco, California, United States of America; 5 Eli and Edythe Broad Center of Regeneration Medicine and Stem Cell Research, University of California San Francisco, San Francisco, California, United States of America; 6 Developmental and Stem Cell Biology Program, University of California San Francisco, San Francisco, California, United States of America; University of California, San Diego, United States of America

## Abstract

The Hypoxia-inducible Factor (HIF) family of transcriptional regulators coordinates the expression of dozens of genes in response to oxygen deprivation. Mammalian development occurs in a hypoxic environment and HIF-null mice therefore die in utero due to multiple embryonic and placental defects. Mouse embryonic stem cells do not differentiate into placental cells; therefore, trophoblast stem cells (TSCs) are used to study mouse placental development. Consistent with a requirement for HIF activity during placental development in utero, TSCs derived from HIF-null mice exhibit severe differentiation defects and fail to form trophoblast giant cells (TGCs) in vitro. Interestingly, differentiating TSCs induce HIF activity independent of oxygen tension via unclear mechanisms. Here, we show that altering the extracellular matrix (ECM) composition upon which TSCs are cultured changes their differentiation potential from TGCs to multinucleated syncytiotropholasts (SynTs) and blocks oxygen-independent HIF induction. We further find that modulation of Mitogen Activated Protein Kinase Kinase-1/2 (MAP2K1/2, MEK-1/2) signaling by ECM composition is responsible for this effect. In the absence of ECM-dependent cues, hypoxia-signaling pathways activate this MAPK cascade to drive HIF induction and redirect TSC fate along the TGC lineage. In addition, we show that integrity of the microtubule and actin cytoskeleton is critical for TGC fate determination. HIF-2α ensures TSC cytoskeletal integrity and promotes invasive TGC formation by interacting with c-MYC to induce non-canonical expression of *Lim domain kinase 1*–an enzyme that regulates microtubule and actin stability, as well as cell invasion. Thus, we find that HIF can integrate positional and metabolic cues from within the TSC niche to regulate placental development by modulating the cellular cytoskeleton via non-canonical gene expression.

## Introduction

Mammalian development occurs in a physiologically hypoxic environment that drives the expression of dozens of genes via the Hypoxia-inducible Factor (HIF) family of transcriptional regulators [1]. A heterodimeric transcription factor composed of alpha and beta subunits, HIF can activate canonical target genes in response to oxygen deprivation by directly binding to hypoxia response elements (HRE) located within their regulatory regions [2], [3]. Due to the short half-life of alpha subunits, HIF activity can be tightly regulated [4]. Mitochondrial oxygen sensing mechanisms produce highly labile reactive oxygen species to ensure that HIF-α subunit stabilization occurs only under hypoxic conditions [5]. Oxygen gradients that are generated as a function of tissue growth can thus activate HIF in a dynamic fashion to pattern the developing embryo [6]. Consistent with this, HIF activity is required for embryonic development [7], [8], [9].

During mammalian gestation, the placenta forms a vital transport interface between the maternal and fetal circulations and its development is also dependent on HIF activity [10], [11], [12], [13]. In mice, two terminally differentiated cell types are primarily responsible for placental function: 1. trophoblast giant cells (TGC) and, 2. multinucleated syncytiotrophoblasts (SynT) [14]. TGCs anchor the placenta to the uterus and direct maternal blood flow to the conceptus while SynTs perform the transport functions of the placenta [15]. The derivation of trophoblast stem cells (TSCs) that differentiate into these placental cells *in vitro* has enhanced our understanding of placental development *in vivo* [16]. Interestingly, however, TSC differentiation using conventional techniques results primarily in the production of TGCs that is associated with the stabilization of HIF-α subunits independent of oxygen tension [10], [17]. We previously demonstrated that HIF induction is critical for TGC differentiation as *Hif-1/2*α ^−/−^ (compound null) or *Hif-1β (Arnt)* ^−/−^ TSCs (collectively referred to as HIF-null) fail to produce TGCs *in vitro*, differentiating primarily into multinucleated SynTs, indicating that HIF activity can suppress cell fusion and SynT fate determination [10], [17]. The mechanisms responsible for differentiation-dependent HIF induction and how HIF ultimately regulates cell fate in the placenta remain unknown, however.

Here, we show that TSC-extracellular matrix (ECM) interactions provide positional cues during normoxia that trigger differentiation-dependent HIF induction via signaling pathways that intersect with metabolic responses to oxygen deprivation. We find that altering the ECM substrate upon which TSCs are cultured impacts differentiation-dependent HIF stabilization and TSC fate. SynT formation is dependent on cell fusion – a process that is associated with significant cytoskeletal reorganization [18], [19] [20], [21]. Consistent with this, HIF-null TSCs exhibit dramatic morphological changes upon syncytialization [17]. We now show that non-canonical HIF-2 activity, induced in response to hypoxia or ECM composition, can prevent this process by promoting *Limk1* expression and subsequent cytoskeletal stabilization.

## Results

### ECM Composition Regulates TSC Fate and HIF Stability Independent of O_2_ Tension

**Figure 1:**
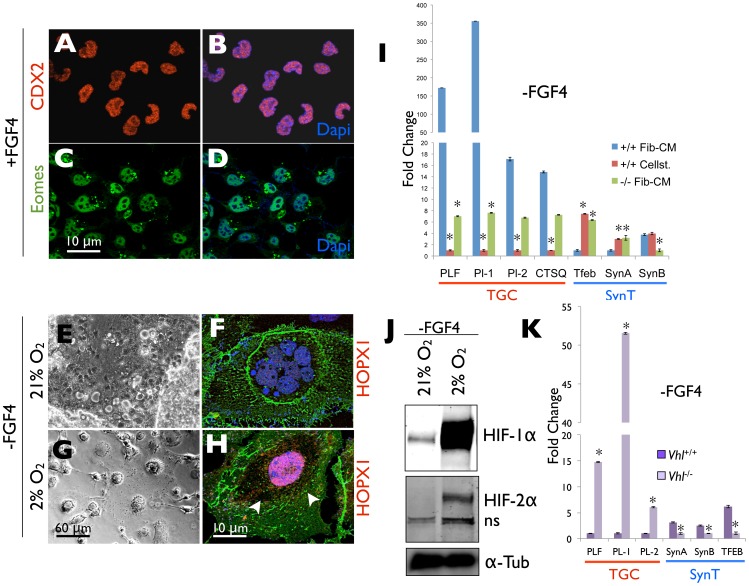
HIF integrates ECM cues and Oxygen Levels to Direct TSC Fate. (A–D) Immunofluorescence microscopy of undifferentiated control TSCs cultured on CELLstart™ with anti-CDX2 and EOMES antibodies (blue = DAPI, red = CDX2, green = Eomes). (E, G) Phase contrast microscopy of control TSCs maintained on CELLstart™ following differentiation for 7 days under normoxic (21% O_2_) or hypoxic (2% O_2_) conditions. (F, H) Immunofluoresce microscopy of control TSCs maintained on CELLstart™ following differentiation for 7 days under normoxic (21% O_2_) or hypoxic (2% O_2_) conditions with an anti-HOPX1 (red) antibody (blue = DAPI). (I) Quantitative RT-PCR analysis of *Pl. I, Pl.II, Ctsq, Plf, Tfeb, SynA* and *SynB* gene expression in wild-type (+/+) TSCs differentiated for 7 days following culture on CELLstart™ or on TC plastic in Fib-CM, compared with *Arnt^−/−^* (−/−) TSCs differentiated following culture on TC plastic in Fib-CM. p values <0.05 versus wild-type Fib-CM indicated by an asterisk. (J) Immunoblot of HIF-1α and -2α protein levels in whole cell lysates of wild-type TSCs differentiated for 7 days following culture on CELLstart at 21%O_2_ or 2% O_2_. (K) Quantitative RT-PCR analysis of *Pl-1, Pl-2, Plf, Tfeb, SynA and SynB* expression in *Vhlh^+/+^* and *Vhlh^−/−^* TSCs differentiated following culture on CELLstart™. p values <0.05 versus wild-type indicated by an asterisk.

**Figure 2:**
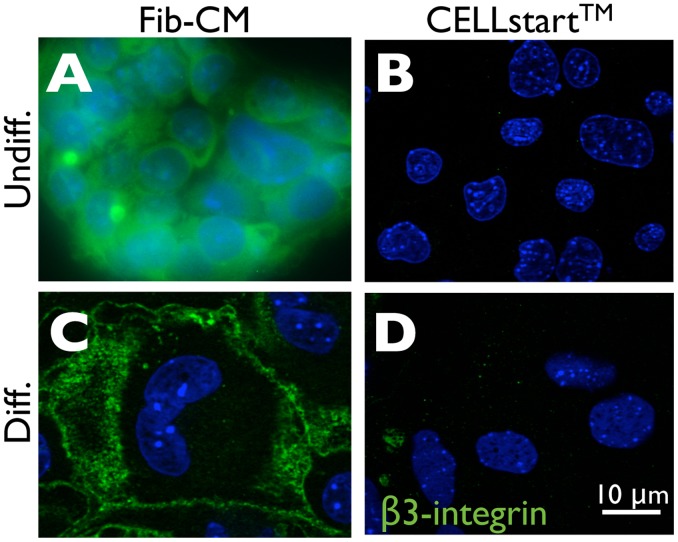
*β*3-Integrin (CD61) is downregulated in TSCs following culture on CELLstart™. Immunofluoresce microscopy of wild-type TSCs maintained on TC plastic in Fib-CM (A) or on CELLstart™ (B) in the presence of FGF4 and heparin (Undiff.) or following differentiation (Diff., C and D) using an anti-CD61 antibody (green) (magnification 630X).

TSC proliferation depends on Fibroblast Growth Factor 4 (FGF4) as well as the presence of fibroblast “feeder” cells or fibroblast conditioned medium (Fib-CM) [16]. In the absence of either, TSCs default to a TGC differentiation program. While screening for culture conditions that could maintain FGF4-dependent TSC growth independent of fibroblasts or Fib-CM, we identified the xeno-free defined ECM substrate, CELLstart™ (Invitrogen) [22], [23]. This ECM substrate is composed primarily of Fibronectin, along with other ECM components [24], and thus represents a physiologically relevant substrate for TSC culture [25], [26]. TSCs maintained on CELLstart™ in the presence of FGF4, but without fibroblasts or Fib-CM, proliferated indefinitely and expressed TSC-specific transcription factors such as CDX2 and EOMES [16](Fig. 1A–D), the levels of which dramatically decreased following FGF4 withdrawal (not shown). Interestingly, however, differentiation in 21% O_2_ (room air) following FGF4 withdrawal of TSCs maintained on CELLstart™ promoted cell fusion and resulted primarily in the formation of multinucleated SynTs (Fig. 1E, 1F), as opposed to the TGCs commonly observed with TSCs maintained on fibroblasts or on TC plastic in Fib-CM [16], [17]. Importantly, differentiation under hypoxic (2% O_2_) conditions could reverse this cell fate choice (Fig. 1G, 1H), blocking SynT formation and generating TGCs expressing the lineage specific transcription factor HOPX1 [27]. Lineage-specific gene expression analyses further confirmed that wild-type TSCs differentiated following culture on CELLstart™ expressed dramatically reduced levels of the TGC-specific markers *Placental lactogens I* and *−2*, *Proliferin* and *Cathepsin Q,* and exhibited increased levels of the SynT markers *Tfeb* and *SynA,* when compared with genetically identical TSCs differentiated following culture on TC plastic in Fib-CM (Fig. 1I). Importantly, this pattern of gene expression was similar to, though more pronounced than, that observed following differentiation of *Arnt^−/−^* or *Hif-1/2*α *^−/−^* TSCs that form SynTs following culture on TC plastic in Fib-CM (Fig. 1I) [10] [17]. We therefore asked whether the alteration of TSC fate following culture on CELLstart™ might be due to impaired HIF-α subunit stabilization that normally occurs during differentiation using standard techniques [17]. Indeed, TSCs differentiated following culture on CELLstart™ in 21% O_2_ failed to stabilize HIF-2α and only slightly accumulated HIF-1α protein levels, whereas differentiation in 2% O_2_ induced both proteins (Fig. 1J). Furthermore, TSCs derived from *Vhlh* ^−/−^ embryos, which exhibit constitutively elevated HIF-1α and -2α due to lack of VHL-dependent ubiquitination and proteasomal degradation [2], [3], still formed TGCs in 21% O_2_ following differentiation despite maintenance on CELLstart™ (Fig. 1K). These results suggest that TSC derivation on fibroblasts and maintenance on TC plastic in Fib-CM provides a set of extracellular cues that promote O_2_-independent HIF stabilization and subsequent TGC formation during differentiation that are lost when TSCs are maintained on the defined ECM substrate, CELLstart™. To understand the mechanisms responsible, we concentrated on cell surface integrin expression, as these molecules play a central role in cell-ECM interactions [28]. TSCs maintained on CELLstart™ were compared with TSCs maintained on TC plastic in Fib-CM. Interestingly, culture on CELLstart™ completely blocked *β*3-integrin expression in both undifferentiated and differentiated TSCs (Fig. 2A–D), suggesting that ECM composition determines cell surface integrin expression and thereby modulates downstream signaling and TSC fate. Importantly, while undifferentiated TSCs maintained on TC plastic in Fib-CM expressed this *β*3-integrin, its cell surface expression was largely restricted to differentiated TGCs, consistent with HIF induction being associated with TGC differentiation.

### ECM- and Oxygen-dependent HIF Stabilization and TGC Formation Occur via MAP2K1/2-dependent Signaling

**Figure 3:**
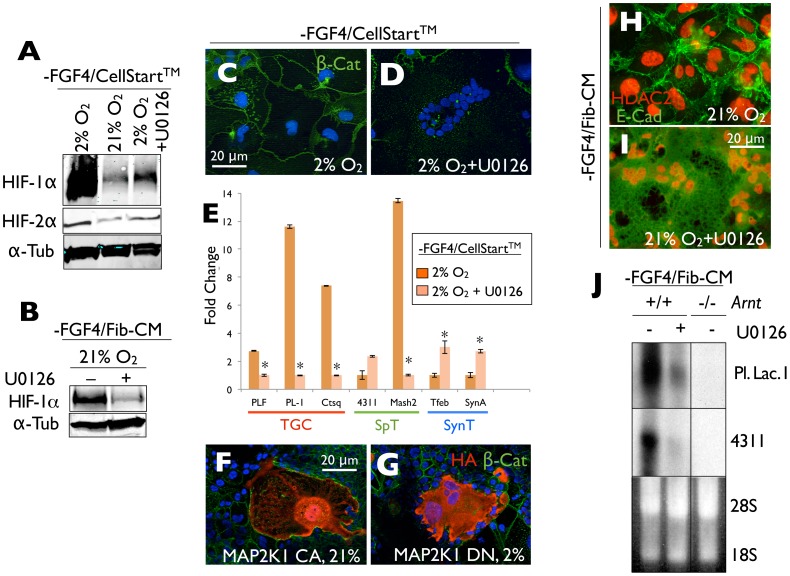
ECM- or oxygen-dependent HIF-α subunit stabilization and TGC formation are dependent on MAP2K1/2 activity. (A) Immunoblot of whole cell lysates obtained from TSCs differentiated in 2% or 21% O_2_, with and without U0126, following culture on CELLstart™, for HIF-1α, -2α or α-Tubulin. (B) Immunoblot of whole cell lysates obtained from differentiated wild-type TSCs following culture on TC plastic in Fib-CM with and without U0126 with a HIF-1α antibody. (C) (D) Immunofluorescence microscopy of TSCs maintained on CELLstart™ following differentiation for 7 days under hypoxic conditions without and with U0126 (10 uM) using anti *β*-Catenin antibodies (green) (blue = Dapi). (E) Quantitaive RT-PCR analysis of *Plf, Pl-I, Ctsq, 4311, Mash2, Tfeb* and *SynA* expression following differentiation of wild-type TSCs cultured on CELLstart™ under hypoxic conditions without and with U0126. p values <0.05 versus drug free control indicated by an asterisk. (F) Immunofluorescence microscopy using anti HA (red) and *β*-Catenin (green) antibodies of control TSCs differentiated following culture on CELLstart™ under 21% O_2_ following transient tranfection with constitutively active HA:MAP2K1 or under (G) 2% O_2_ following transient transfection with dominant negative HA:MAP2K1. (H, I) Immunofluorescence microscopy of wild-type TSCs differentiated following culture on TC plastic in Fib-CM in 21% O_2_ with and without U0126 with antibodies for HDAC2 (red) and E-Cadherin (green). (J) Northern blot analysis of lineage specific marker gene expression in wild-type TSCs maintained on TC plastic in Fib-CM and differentiated with and without U0126, compared with differentiated *Arnt* ^−/−^ TSCs.

We suspected that candidate pathways capable of integrating ECM-dependent signals with oxygen-dependent inputs might include members of the mitogen activated protein kinase (MAPK) cascade. This is due to the fact each set of stimuli can independently activate this signaling cascade [29] [30], [31], [32], [33]. Consistent with this, pharmacological inhibition of MAP2K1/2 (MEK-1/2) activity prevented HIF-α subunit stabilization during hypoxic (2% O_2_) differentiation of TSCs cultured on CELLstart™ (Fig. 3A) and during normoxic (21% O_2_) differentiation following culture on TC plastic in Fib-CM (Fig. 3B). Furthermore, hypoxic TGC formation of TSCs following culture on CELLstart™ could be suppressed with the same MAP2K1/2 inhibitor (Fig. 3C–E), while transient expression of constitutively active MAP2K1 (Fig. 3F) promoted TGC formation under normoxic conditions and dominant negative MAP2K1 allowed cell fusion under hypoxic conditions (Fig. 3G). And finally, pharmacological MAP2K1/2-inhibition could prevent TGC formation and promoted SynT differentiation in TSCs that had been cultured on TC plastic in Fib-CM (Fig. 3H, 3I). Northern blot analysis confirmed that MAP2K1/2 inhibition in wild-type TSCs suppressed expression of the TGC as well as spongiotrophoblast (SpT) marker genes, *Placental lactogen I* and *4311*, respectively, nearly to levels observed in differentiated *Arnt^−/−^* TSCs (Fig. 3J). Collectively, these results confirm that ECM- and oxygen-dependent HIF-α subunit stabilization and subsequent TGC formation occurs through a MAP2K1/2-dependent pathway.

### Cytoskeletal Rearrangement is Central to MAP2K1/2-mediated TGC Formation

**Figure 4:**
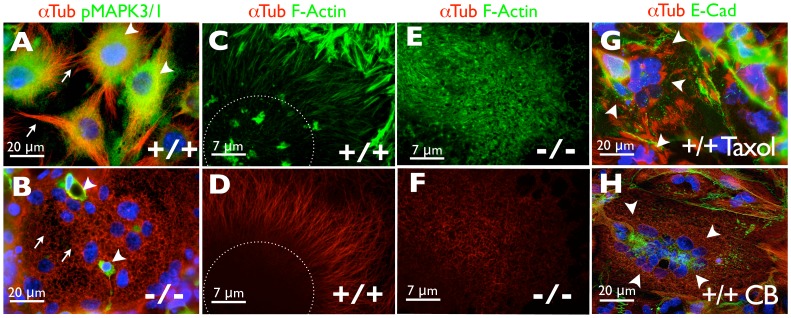
MAP2K1/2 inhibition and cytoskeletal rearrangement in differentiating HIF-null TSCs. Immunofluorescence microscopy of terminally differentiated wild-type (+/+) TGCs (A) and *Hif-1/2*α^−/−^ (−/−) SynTs (B) with an anti α-Tubulin (red), or p-MAPK3/1 (green) antibody (arrows = microtubules, arrowheads = pMAPK3/1). (C) Confocal microscopy imaging of polymerized actin via FITC-phalloidin staining (green) or (D) α -Tubulin (red) in terminally differentiated control TGCs (dashed line indicates approximate location of nucleus). (E) Confocal microscopy imaging of polymerized actin via FITC-phalloidin staining (green) or (F) α-Tubulin (red) in terminally differentiated HIF-null SynTs. (G) Differentiation of control TSCs in the presence of Taxol (G) or (H) Cytochalasin B (CB) promoted the formation of multinucleated cells (arrowheads) following culture on TC plastic in Fib-CM. α-Tubulin (red) and E-Cadherin (green).

Similar to mitochondrial responses to changing O_2_ levels, dynamic integrin ligation in response to changes in ECM composition allow a cell to sense its environment by converting positional information into downstream signals [34]. These frequently result in cytoskeletal reorganization [35] that can promote cell migration or other alterations in cell behavior [36] **.** Additionally, trophoblast differentiation has been associated with significant cytoskeletal changes [19], [20], [37]. We therefore examined the cytoskeletal organization of differentiated control and *Hif-1/2*α*^−/−^* TSCs that had been maintained on TC plastic in Fib-CM and analyzed its association with MAP2K1/2 activation. TGCs derived from control TSCs contained robust MTs extending the length of the cell (Fig. 4A, 4D), while multinucleated SynTs derived from *Hif-1/2*α*^−/−^* TSCs exhibited a disrupted microtubule (MT) network consisting of “broken” appearing MT fragments (Fig. 4B arrows, 4F). MT integrity associated strongly with MAP2K1/2 activity, as control TGCs with robust MTs stained strongly for the phosphorylated versions of the MAP2K1/2 target MAPK3/1 (ERK-1/2) (Fig. 4A), while multinucleated SynTs derived from *Hif-1/2*α*^−/−^* TSCs did not (Fig. 4B). Only unfused SynT progenitors that did not contain “broken” MTs continued to exhibit the active form of this kinase in *Hif-1/2*α*^−/−^* TSCs (Fig. 4B, arrowheads). Additionally, we observed dramatic differences in the actin cytoskeleton, with robust stress fibers noted in TGCs (Fig. 4C) while SynTs exhibited a disorganized actin cytoskeleton containing high amounts of diffusely distributed F-actin (Fig. 4E). To formally test whether cytoskeletal integrity could regulate TSC fate, we investigated whether pharmacological MT or actin disrupting agents could promote SynT formation of TSCs cultured using conventional techniques. Indeed, the MT disrupting agent Taxol (Paclitaxel) (Fig. 4G) and the actin disrupting agent cytochalasin B (Fig. 4H) inhibited TGC formation and promoted the formation of multinucleated SynTs in control TSCs that had been maintained on TC plastic in Fib-CM.

### HIF-dependent *Limk1* Expression Promotes TGC Differentiation

**Figure 5:**
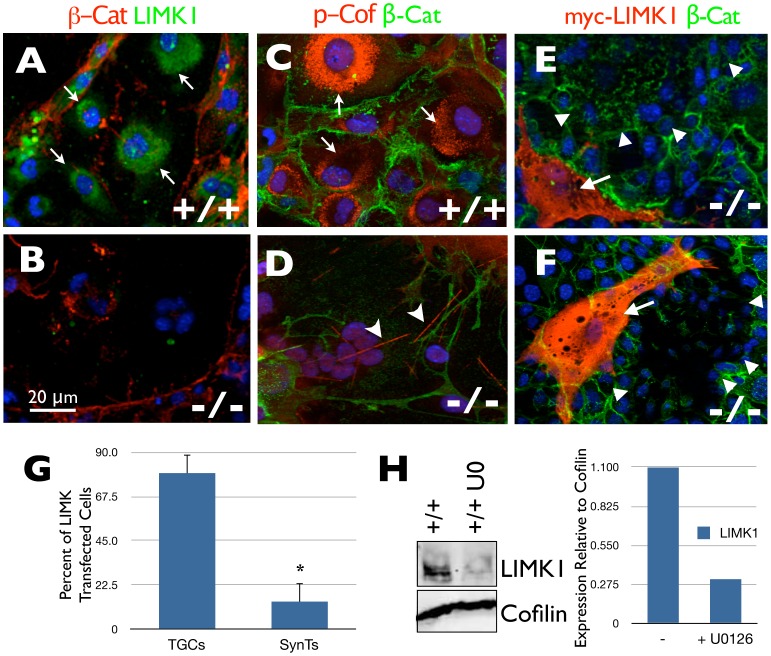
HIF-dependent LIMK1 expression promotes TGC formation in TSCs. Immunofluorescence microscopy of terminally differentiated control TGCs (A) and *Hif-1/2*α^−/−^ SynTs (B) with a *β*-Catenin (red) and LIMK1 (green) antibody (arrows = perinunclear LIMK1 staining). Immunofluorescence microscopy of terminally differentiated control TGCs (C) and *Hif-1/2*α^−/−^ SynTs (D) with a *β*-Catenin (green) and p-Cofilin (red) antibody (arrows = perinunclear p-Cof staining, arrowheads = cofilin rods). (E, F) Two representative images of TGC formation (arrows) following transient myc-LIMK1 expression in *Hif-1/2 ^−/−^* TSCs while untransfected cells primarily form SynTs (arrowheads) (red = myc-LIMK1, green = *β*-catenin). (G) Quantification of the percentage of LIMK1 transfected HIF-null TSCs differentiated into TGCs vs. SynTs. (H) Immunoblot analysis of LIMK1 levels in differentiated wild-type (+/+) TSCs without and with U0126 (U0). Integrated densitometry confirmed the decreased expression of LIMK1, relative to total Cofilin, in control TSCs differentiated in the presence of U0126.

We next focused our efforts on identifying cytoskeleton regulatory molecules that may be misregulated in the absence of HIF activity. Gene array studies indicated that expression of the gene encoding Lim domain kinase 1 (LIMK1), an enzyme responsible for regulating MT and Actin integrity [38], was significantly downregulated in the absence of HIF activity (not shown). Immunofluorescence microscopy confirmed that HIF-null TS cells differentiated into SynTs expressed no detectable LIMK1 expression, while control TGCs expressed robust LIMK1 protein levels in a perinuclear distribution (compare Fig. 5A and 5B). Immunoblot analyses confirmed this (see below) and indicated that the MAP2K1/2 inhibitor U0126 significantly decreased LIMK1 levels in wild-type TSCs differentiated following maintenance on TC plastic in Fib-CM (Fig. 5H), consistent with its ability to block HIF stabilization during differentiation. Furthermore, we found that the LIMK1 target Cofilin was highly phosphorylated, also in a perinuclear distribution, and therefore inactive in TGCs (Fig. 5D), but not in SynTs (Fig. 5E), which frequently contained prominent Cofilin rods that form when Cofilin is hyperactive [39] (Fig. 5E, arrowheads). The residual phosphorylation of Cofilin rods in SynTs may be due to their residual low-level LIMK2 levels (see below) [38]. Furthermore, transient LIMK1 expression in differentiating *Hif-1/2*α*^−/−^* TSCs promoted the appearance of large TGCs in the majority of transfected cells, as opposed to the SynTs commonly observed following their differentiation (Fig. 5F, 5G). These results suggest that HIF-dependent LIMK1 expression can regulate TSC fate downstream of ECM- or oxygen-dependent MAP2K1/2 activation by modulating the cytoskeleton.

### Non-Canonical HIF-2α Activity Drives TSC Fate via LIMK1-mediated Cytoskeletal Stabilization

**Figure 6:**
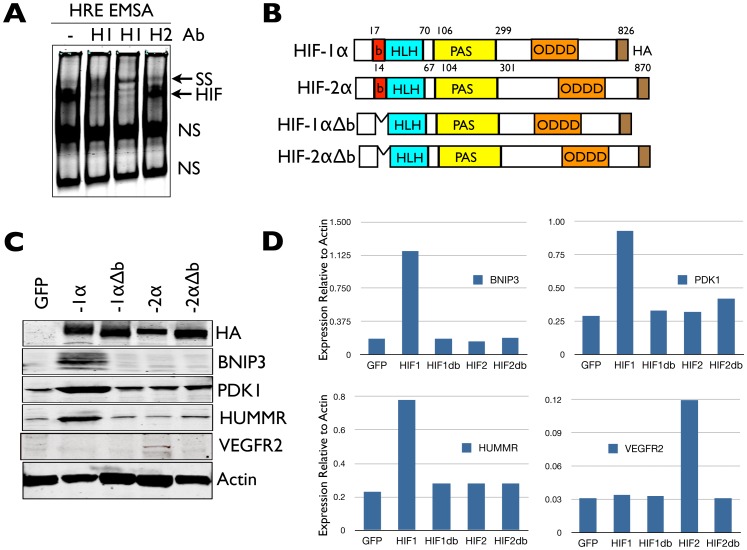
Canonical vs non-canonical HIF target gene-expression in TS cells. (A) Electrophoretic mobility shift assay (EMSA) of differentiated control TGC nuclear extracts with and without 2 different anti-HIF-1α antibodies (H1) or a HIF-2α antibody (H2) (“supershift” SS, NS, non-specific complexes.) (B) Schematic representation of full-length HIF-1α and HIF-2α, as well as versions lacking their DNA binding basic (b) domains (HLH, Helix-loop-helix, PAS, Per-Arnt-Sim, ODDD, oxygen-dependent degradation domain). (C) Immunoblot detection of stable HA-epitope tagged HIF-1α, HIF-1αΔb, HIF-2α and HIF-2αΔb protein, as well as respective target gene protein products in *Hif-1/2*α^−/−^ TSCs. (D) Integrated densitometric quantification of HIF target gene protein products relative to Actin expression in each respective cell line.

We next investigated how HIF regulates *Limk1* gene expression during TSC differentiation. We first determined whether we could detect canonical HIF-DNA interactions in differentiated TSCs via electrophoretic mobility shift assays (EMSA). As seen, control TSCs maintained on TC plastic in Fib-CM and differentiated in room air (21% O_2_) contained abundant HRE-bound HIF complexes (Fig. 6A), consistent with our prior observations of HIF activation during normoxic differentiation of TSCs maintained using conventional methods (13). Interestingly, however, while two different HIF-1α-specific antibodies produced a “supershift, SS” in differentiated TSC nuclear extracts, a HIF-2α-specific antibody did not (Fig. 6A), suggesting that HIF-1α was predominantly responsible for canonical HRE-mediated gene expression in differentiating TSCs. To interrogate the requirement for direct HIF-DNA binding during TSC fate determination, we stably reconstituted *Hif-1/2*α^−/−^TSCs [10] utilizing a PiggyBac transposon system [40] either with HA-tagged wild-type HIF-1α or HIF-2α individually, or with mutant forms of each lacking their DNA binding basic domains (HIF-1αΔb, HIF-2αΔb) (Fig. 6B). Deletion of the basic domain prevents HRE binding and canonical target gene expression by HIF complexes without affecting their stability [41]. As expected, full-length versions of each were capable of activating their respective canonical downstream target genes in HIF-null TSCs, while versions lacking their basic domains could not (Fig. 6C, 6D).

**Figure 7:**
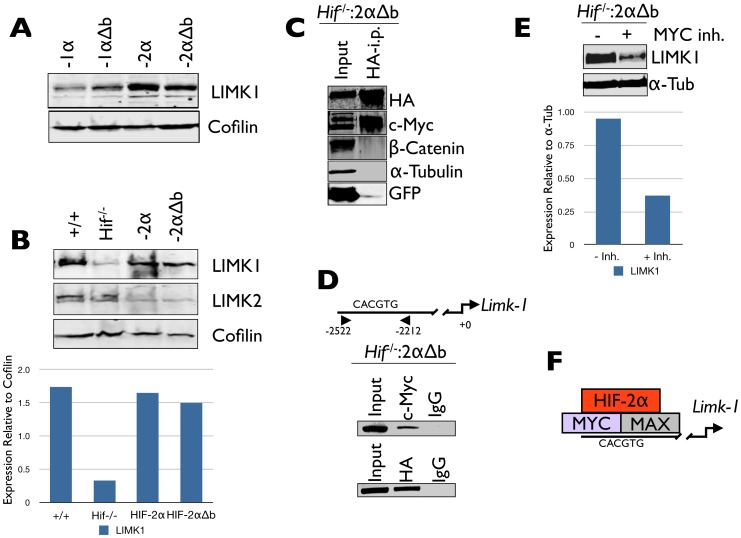
Canonical target gene-independent HIF-2 activity drives LIMK1 expression in TS cells via c-MYC interaction. (A) Immunoblot analysis of LIMK1 protein levels in *Hif-1/2*α^−/−^ TSCs stably reconstituted with full length HIF-1α or -2α, as well as versions lacking their basic domains. (B) Immunoblot analysis of LIMK1 and LIMK2 expression in control (+), *Hif-1/2*α^−/−^ (*Hif* ^−/−^), and HIF-2α and HIF-2αΔb reconstituted *Hif-1/2*α^−/−^ TSCs. Integrated densitometric analysis confirmed that both HIF-2α, as well as HIF-2αΔb, restored LIMK1 expression to control levels in *Hif-1/2α* ^−/−^ TSCs. (C) Immunoprecipitation with an anti-HA antibody of HA-tagged HIF-2αΔb followed by immunoblot with anti-HA, c-MYC, *β*-Catenin, α-Tubulin and GFP antibodies. (D) Schematic representation of E-box element identified within the *Limk1* promoter. Chromatin immunoprecipitation (ChIP) analysis indicated specific binding of c-MYC and HA-tagged HIF-2αΔb to this element. (E) Immunoblot analysis of LIMK1 protein levels in HIF-2αΔb expressing *Hif-1/2*α*^−/−^* TSCs without (-) or with (+) c-MYC inhibitor. Integrated densitometric analysis confirmed reduced expression of LIMK1 relative to α-Tubulin in drug treated cells. (F) Schematic representation of HIF-2α interacting with MYC:MAX heterodimers at the *Limk1* promoter.

**Figure 8:**
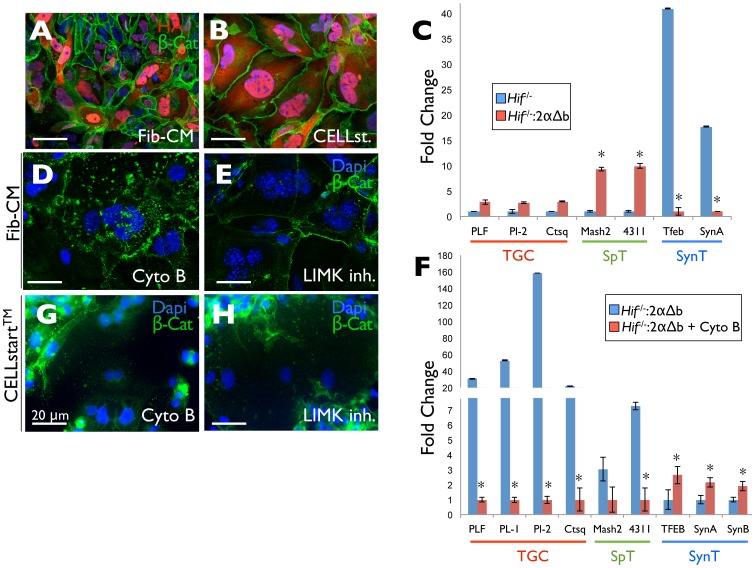
Non-canonical HIF-2-dependent LIMK1 expression promotes TGC formation. Immunofluorescence microscopy of HIF-2αΔb expressing *Hif-1/2*α*^−/−^* TSCs differentiated following culture on TC plastic in Fib-CM (A) or on CELLstart™ (B) (green = *β*-Cat, red = HA). (C) qRT-PCR-based comparison of expression levels of the TGC (PLF, Pl-2, Ctsq), spongiotrophoblast (Mash2, 4311) and SynT (Tfeb, SynA) markers in *Hif-1/2α−/−* and HIF-2αΔb reconstituted HIF-null (*Hif^−/−^:2*αΔ*b)* TSCs differentiated for 7 days following culture on TC plastic in Fib-CM. p values <0.05 versus HIF-null indicated by an asterisk. (D) Immunofluorescence microscopy of HIF-2αΔb expressing *Hif-1/2α^−/−^* TSCs differentiated following culture on TC plastic in Fib-CM in the presence of Cytochalasin B or (E) the LIMK inhibitor (BMS-5 10 uM)(green = *β*-Cat). (F) qRT-PCR-based comparison of expression levels of the TGC (PLF, Pl-1, Pl-2, Ctsq), spongiotrophoblast (Mash2, 4311) and SynT (Tfeb, SynA) markers in HIF-2αΔb reconstituted HIF-null (*Hif^−/−^:2*αΔ*b)* TSCs differentiated for 7 days following culture on TC plastic in Fib-CM without and with the actin cytoskeleton disrupting agent cytochalasin B (Cyto B). p values <0.05 versus drug free control indicated by an asterisk. (G) Immunofluorescence microscopy of HIF-2αΔb expressing *Hif-1/2*α*^−/−^* TSCs differentiated following culture on CellStart™ in the presence of Cytochalasin B or (H) the LIMK inhibitor (BMS-5 10 uM)(green = *β*-Cat).

Using this system, we investigated the HIF-α subunit dependence of LIMK1 expression. Interestingly, while neither HIF-1α nor HIF-1αΔb restored LIMK1 protein levels in *Hif-1/2*α*^−/−^* TSCs to that observed in wild-type TSCs, both HIF-2α and HIF-2αΔb did (Fig. 7A, 7B), suggesting it to be a non-canonical HIF-2-specific target gene. We suspected that the HIF-2-specificity may be due to its known interaction with and activation of c-MYC-dependent transcription [42], [43]. To test this, we immunoprecipitated HA-tagged HIF-2αΔb in reconstituted *Hif-1/2*α *^−/−^* TSCs and detected strong c-MYC interaction (Fig. 7C). We next identified a 100% conserved c-MYC binding E box (CACGTG) in the *Limk1* promoter, but no canonical HIF binding sites, and detected both c-MYC as well as HIF-2α binding to this site *in situ* in differentiated TGCs by chromatin immunopreciptation (Fig. 7D). We then tested whether pharmacological c-MYC inhibition could block non-canonical HIF-2α-dependent *Limk1* gene expression, and found that it could decrease LIMK1 protein levels in HIF-2αΔb expressing cells (Fig. 7E). This indicates that HIF-2α interacts with c-MYC containing transcriptional complexes during TSC differentiation and that this interaction contributes to *Limk1* expression during TGC differentiation (Fig. 7F). Stable HIF-2αΔb expressing HIF-null TSCs, whether cultured on CELLstart or on TC plastic in Fib-CM, failed to fuse into multinucleated SynTs and differentiated along the TGC lineage (Fig. 8A, 8B, 8C). Importantly, pharmacological cytoskeleton disruption (Fig. 8D, 8F, 8G) could reverse this effect, promoting SynT formation in these cells. Additionally, pharmacological LIMK1 inhibition [44], [45] promoted the formation of multinucleated cells (Fig. 8E, 8H) in HIF-null TSCs reconstituted with HIF-2αΔb.

## Discussion

**Figure 9:**
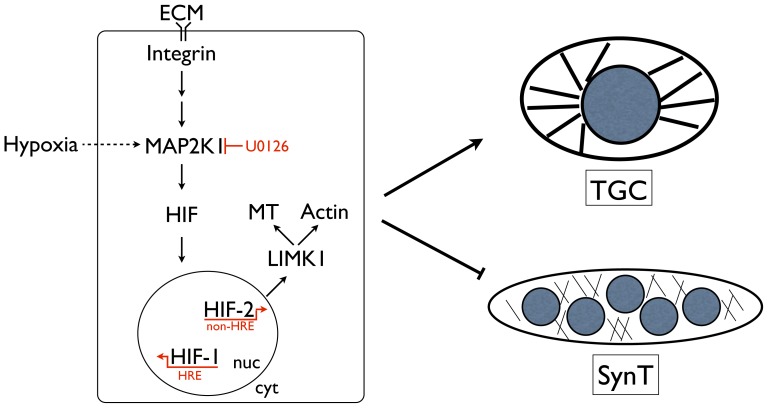
Model of HIF-dependent integration of positional and metabolic cues in the TSC niche. ECM composition regulates HIF stabilization likely downstream of cell surface integrin ligation via MAP2K1/2 activation. Inside-out integrin signaling mechanisms may also be operative. Oxygen sensing and signaling pathways intersect with this signaling cascade to stabilize HIF, when ECM-dependent cues are absent. Stabilized HIF can act via canonical and non-canonical target genes. Non-canonical HIF-2, by interacting with MYC:MAX heterodimers, bind the *Limk1* promoter to activate its expression. LIMK1 promotes microtubule and actin stability, critical for TGC formation, and thereby prevents SynT formation. HIF, therefore, can integrate divergent environmental inputs from within the placenta to regulate cell fate via non-canonical gene expression.

Collectively, our results indicate that oxygen- and canonical target gene-independent HIF activity can drive TSC fate in response to positional cues encoded by ECM components within the TSC microenvironment (Fig. 9). Initially thought to function as a mere scaffold, the ECM is now known to regulate many aspects of cell behavior, including proliferation and growth, survival, migration, and differentiation [28], [46]. Primary components of the ECM are structural proteins (e.g., collagens, laminins, fibronectin, vitronectin and elastin) and specialized glycoproteins that can interact with molecules having important biological functions such as growth factors. The precise composition varies by location. In the stem cell niche, the ECM can provide instructive cues for cell fate decisions via the integrin family of heterodimeric cell surface receptors [26], [47], [48]. In erythropoiesis, for example, adhesion of primary erythroid progenitors to fibronectin mediated by α_4_ *β*_ 1_ integrin is necessary for proper proliferation *in vitro* [49]. In this system, signals from the ECM cooperate with signals from the soluble factor erythropoietin to activate pathways necessary for terminal differentiation and proliferation. In TSCs, altering their ECM in this way alters their cell surface *β*3-integrin expression, and subsequent HIF induction during differentiation. We have shown here that in TSCs, altering their ECM in this way alters their cell surface *β*3-integrin expression, and subsequent HIF induction during differentiation. Supporting a link between HIF activity and ECM-dependent integrin ligation, αv*β*3 activation can trigger HIF accumulation in some cancer cells [50] and HIF-deficiency negatively affects TSC surface *β*3-integrin localization [51], suggesting that HIF-alpha subunit stability can both be activated by, as well as further promote, cell surface integrin activity. Importantly, trophoblast adhesion to ECM is governed by integrins [52]–[53] and hypoxic conditions promote trophoblast invasion in utero [54].

The mitogen activated protein kinase (MAPK) cascade is a frequent point of convergence from multiple environmental inputs [35], [55]. We therefore reasoned that candidate signaling pathways responsible for oxygen-dependent HIF induction that could interface with ECM-dependent signaling would include members of this family. The classical extracellular regulated kinase (ERK)/MAPK signaling pathway is an obvious candidate because it: 1. Regulates cell fate in a broad range of organisms [56], [57], [58], [59]; 2. Responds to hypoxia downstream of mitochondrial ROS [60] and, 3. Is activated by integrin ligation [61]. Importantly, in mice, MEK1 (renamed MAP2K1), as well as its downstream target ERK2 (renamed MAPK1), are critical regulators of placentation [62], [63], [64], [65]. Interestingly, MAP2K1 deficiency results in placental malformation characterized by an excess of multinucleated cells within affected placentas *in vivo* [66], similar to the formation of multinucleated SynTs with HIF-deficient TSCs *in vitro*, while genetic disruption of the MAP3K B-Raf results in placental malformation associated with diminished HIF-1α protein levels [67], consistent with our results.

Our results also shed novel insights into HIF-dependent cell fate determination. We found that HIF-2αcan interact with c-MYC to enhance *Limk1* gene expression and promote cytoskeletal integrity, thereby enhancing TGC differentiation, via non-canonical means. While HIF-2α has previously been shown to activate c-MYC-dependent target genes [42], [43], to our knowledge this is the first demonstration of a role for this mechanism during normal development. Importantly, c-MYC is required for normal placentation in mice [68]. Also, while HIF activity is generally assumed to result from the reduced oxygen tension frequently encountered during development, our results provide evidence that ECM composition can be added to a growing list of O2-independent factors that can also drive HIF-dependent developmental programs such as Insulin-like Growth Factor 1 [69], or Runx2-mediated HIF stabilization [70]. Additionally, we show that the dramatic cytoskeletal changes observed in SynTs are not a simple by-product of cell fusion, but help regulate cell fate decisions in the placenta. LIMK1 is likely activated downstream of Rho kinase which is known to be induced during TGC formation [37]. Interestingly, HIF stability can be influenced by MT integrity [71], [72], suggesting the possibility of a feed-forward mechanism whereby MAPK-dependent HIF activity promotes MT integrity, which further enhances HIF stability during TGC differentiation.

In addition to playing important roles during normal placental development, the pathway outlined here is likely to be involved in pregnancy complications such as preeclampsia wherein fetal trophoblasts fail to properly invade maternal tissues. While aberrant oxygenation and HIF activity have previously been associated with preeclampsia [73], [74], its ability to regulate LIMK1 has particular relevance, given that LIMK1 has recently been shown to be important for tumor cell invasion in humans [44] as well as collective cell migration in *D. melanogaster* [75]. Furthermore, in addition to altered hypoxia signaling, ECM remodeling is also frequently disrupted in PE [76], suggesting novel roles for HIF in linking these disparate processes. Further investigation of the intersection of these pathways by HIF activation should therefore yield novel insights into the etiology of this intractable syndrome.

## Methods

### TSC Culture

Mouse TSCs were derived on human placental fibroblasts as previously described [17]. Prior to differentiation experiments, TSCs were subjected to a differential plating to remove fibroblasts and subsequently cultured for two passages in 70% Fib-CM with FGF4 and heparin [16]. Differentiations were performed in standard TSC medium without FGF4 or heparin, on tissue culture treated plates or 0.2% gelatin coated glass coverslips, for 7 days. *Vhlh* ^+/−^ and ^−/−^ TSCs were generously provided by M. Celeste Simon (U. Penn). Hypoxia (2% O_2_) was produced with the Biospherix XVivo incubator. For differentiation in the presence of inhibitors, TSCs were cultured in plain TSC medium containing either 10 uM U0126 (Pierce Biotechnology, Rockford, IL), 5 uM Paclitaxel (Taxol, Calbiochem), 10 ug/ml Cytochalasin B (Sigma), 60 uM c-MYC inhibitor (Sigma) or 10 uM LIMK inhibitor BMS-5 (Synkinase).

### Adaptation of TSCs on CELLstart™

For the adaptation of TSCs to the xeno-free substrate CELLstart™ (Invitrogen), cells were passaged from feeder culture using mild trypsinization and plated on CELLstart™ coated tissue culture dishes according to manufacturer’s instructions. Cells were passaged in TSC medium with FGF4 and heparin, but without Fib-CM, upon reaching approximately 75% confluence. Following 5–6 passages, TSC lines on CELLstart™ exhibited a distinct morphology and maintained that morphology for greater than 20 generations in the absence of Fib-CM.

### Northern Blot

Northern blot hybridization was performed with the probes for placental lactogen I and 4311 as described previously [17].

### Immunoblotting and Immunofluorescence Staining

Whole cell lysates were prepared using a buffer consisting of 150 mM NaCl, 50 mM Tris-HCl (pH 7.4), containing 1 mM EGTA, 1 mM EDTA, 1% Triton X-100, 1% SDS and 10% glycerol. TSCs were incubated with PHEMT buffer (60 mM Pipes, 25 mM Hepes, ph 6.9, 10 mM EGTA, 4 mM MgCl_2_ and 0.5% Triton X-100) for 30 min (4°C) and centrifuged to fractionate TSCs into soluble (supernatant) and insoluble fractions (pellet). Immunoblotting was performed with ECL (Amersham) or Odyssey Western blot methodologies and analyzed by Odyssey infrared imaging system (LI-COR Biosciences, Lincoln, NE) using the appropriate secondary antibodies. Briefly, 50–100 ug of whole cell lysates were run on 7.5–12% SDS-PAGE gel, transferred on PVDF membrane, applied with the primary antibody as indicated. For immunoprecipitation, 500 ug of whole cell lysates were immunoprecipitated with the indicated antibody and immunoblotted. The following antibodies were used for immunoblotting, EMSA, immunoprecipitaion and immunofluoresence staining: CDX2 (Biogenex), EOMES (Orbigen), anti human/mouse HIF-1α (R&D Systems, Minneapolis, MN), HIF-1α c-terminal (Cayman Chemical, Ann Arbor, MI), HIF-2α NB 100–122 (Novus Biologicals), ARNT 2B10 (Abcam, Cambridge, MA), pMAPK3/1 (pERK; Cell Signaling, Danvers, MA), MAPK1(ERK2; Epitomics, Burlingame, CA), α-Tubulin (NeoMarkers, Fremont, CA), Ac- α-Tubulin (Sigma-Aldrich), LIMK1 (BD Biosciences), LIMK2 (Proteintech), Cofilin (BD Biosciences), p-Cofilin (CellSignal), PDK1 (StressGen), BNIP3, VHL M-20, HOPX1 (Santa Cruz Biotechnology, Santa Cruz, CA), *β*-catenin (Cell Signaling), c-MYC (SCBT), HA (Zymed, South San Francisco, CA), GFP (Aves), E-cadherin (BD Transduction Pharmingen), and HDAC2 (Zymed). Integrated densitometric analysis was performed using Adope Photoshop.

### Immunoprecipitation

Adherent cells were washed twice by addition of ice cold PBS to the monolayer and disposal of the supernatant. 1 ml of freshly made ice cold lysis/was buffer (50 mM Tris-HCl, 150 mM NaCl pH 7.5, 1% Nonidet P40 0.5% sodium deoxycholate supplemented with 1 complete tablet from Roche) was added to the washed cell monolayers to achieve a concentration of 10^6^–10^7^ cells/ml. Cells were scraped into an eppendorf, and sonicated on ice with 5 pulses each for 8 seconds. Lysate was spun down at 13000 rpm for 5 minutes. Supernatant (except 200 ul) was put onto a new tube. The un-lysed pellet was resuspended into the 200 ul remaining lysate, and sonicated again, the tube centrifuged at 13000rpm for 5 minutes and the new lysate added to the original lysate. 50 ul of this lysate was kept aside as input. To reduce background a pre-clearing step was performed overnight. 50 ul of the homogeneous protein G- agarose (Roche) suspension, equilibrated in the lysis buffer, was added to the 1 ml lysate at 2–8°C on a rotating platform overnight. Beads were then pelleted by centrifugation at 2000×g for 2 minutes at 4°C. Supernatant was transferred to a new tube. 50 ul of Agarose-coupled chicken anti-HA (Aves Labs, Inc. Oregon) was equilibrated in the wash/lysis buffer, centrifuged for 2 minutes at 2000×g, and supernatant discarded. The cell lysate was added to these beads and rotated (gentle end-over-end mixing) overnight at 4°C. The lysate/bead complex was then centrifuged for 2 minutes at 2000×g. Pellet was washed 4× by resuspending in lysis/wash buffer. A final wash was performed once for 30 minutes. Beads were then resuspended in 90 ul of 2× SDS sample buffer, boiled for 10 minutes at 95°C. Beads were collected by centrifugation at 2700×g for 2 minutes at 4°C and SDS-PAGE performed with the supernatant.

### CHIP Assay

Cells were washed with D-PBS and cross-linked by 1% formaldehyde (37 wt% from Sigma-Aldrich) for 10 min at 37°C. Glycine (2.5M) was added and incubated in room temperature for 10 minutes. Cells were then washed 3× in D-PBS for 5 minutes and harvested with D-PBS in the presence of protease inhibitor (EDTA-free Complete, Roche Applied Science). These cells were then centrifuged at 3000 rpm for 5 minutes and lysis buffer (1% SDS, 10 mM EDTA, 50 mM Tris-HCl, PH 8.1, with fresh protease inhibitor) was added, sonicated and incubated overnight at 65°C. Rnase A was added and incubated at 37°C, after which 2 ul 0.5 M EDTA, 4 ul 1 M Tris-HCL PH 8.1, 1 ul proteinase K was added and incubated for 2 hours at 45°C to generate 200- to 500-bp DNA fragments, which were subsequently confirmed by agarose gel electrophoresis. Pre-clearing was performed by using 50 ul protein G Sepharose (washed in dilution buffer, 0.01% SDS, 1.1% Triton X-100, 2 mM EDTA, 20 mM Tris-HCl, PH 8.1, 150 mM MgCl2), 30 ul normal IgG, 20 ug salmon sperm and rotated for 2 hours at 4°C. 20 ul was taken as input. Protein G sepharose and antibody complexes were prepared by re-suspending protein G with dilution buffer, 1 ug antibody and incubated on a rotator at 4°C overnight, and then washed twice with dilution buffer and centrifuged at 3000 rpm for 1 minute. Precleared samples were added to the antibody complex beads and rotated at 4°C overnight to collect antibody/antigen/DNA complex. Protein G complex was centrifuged at 3000 rpm for 1 minute, and supernatant removed. Protein G complex was washed sequentially for 5 minutes twice with: low salt buffer (0.1% SDS, 1% Triton X-100, 2 mM EDTA, 150 mM NaCl, 20 mM Tris-HCl, PH 8.1), high salt buffer (0.1% SDS, 1% Triton X-100, 2 mM EDTA, 500 mM NaCl, 20 mM Tris-HCl, PH 8.1), LiCl buffer (0.25 M LiCl, 1% NP-40, 1% deoxycholate, 1 mM EDTA, 10 mM Tris-HCl, PH 8.1), with TE buffer 1 mM EDTA, 10 mM Tris-HCl, PH 8.1). 200 ul of elution buffer was used containing 20 ul 10% SDS, 20 ul 1 M NaHCO3, 160 ul H2O. 100 ul of elution buffer was added to each tube containing the agarose/antibody complex or the input and incubated in room temperature for 15 minuites. Agarose complex was pelleted by centrifugation (5000×g, 1 minute) and supernatant collected. This was repeated with another 100 ul elution buffer, and added to the first eluate (total volume 200 ul). Protein/DNA complexes were reversed to free DNA by adding 8 ul 5 M NaCl, 1 ul 10 mg/ml RNase A, and incubated at 65°C overnight. DNA was purified by using spin columns from Qiagen. Before purification, 4 ul 0.5 M EDTA, 8 ul 1 M Tris-HCl, and 1 ul protein kinase k was added to each tube and incubated at 45°C for 1 hour. Specifically bound purified DNA fragments were visualized by PCR using specific primers: (forward) 5′-tgcatgcaccctaaataaaaata-3′, (reverse) 5′- ccttgaggagcacacataaccat-3′.

### mRNA Expression Analysis by Real Time PCR

RNA was extracted using TRIzol® Reagent (Invitrogen), and isolation was carried out according to manufacturer’s instructions. 2 mcg of RNA per sample was made into cDNA using MMLV reverse transcriptase (Applied Biosystems). Prepared cDNA was amplified using SYBR® Green PCR Master Mix (Life Technologies) and the Bio-Rad iCycler iQ multicolor real time PCR detection system. Cycle threshold (Ct) values were normalized for amplification using *Hypoxanthine guanine phosphoribosyl transferase* (*Hprt*). Data analysis for real time quantitative PCR was done using the deltaCt method. Primer sequences are as follows: *Prolilferin* (*Plf*) Sense – tgaggaatggtcgttgcttt, antisense – tctcatggggcttttgtctc. *Placental Lactogen 1* (*Pl-1*) Sense – tggtgtcaagcctactccttt, Antisense – caggggaagtgttctgtctgt. *Placental Lactogen 2* (*Pl-2*) Sense – ccaacgtgtgattgtggtgt, Antisense – tcttccgatgttgtctggtg. *Hypoxanthine guanine phosphoribosyl transferase* (*Hprt*) Sense –aaacaatgcaaactttgctttcc, Antisense – ggtccttttcaccagcaagct. *Syncitin A* (SynA) Sense – tactcctgcccgatagatga, Antisense – ccgtttttcttaacagtgggt. *Syncytin B (SynB)* Sense – ccaccacccatacgttcaaa, Antisense – ggttatagcaggtgccgaag. *Transcription factor EB (Tfeb)* Sense – aacaaaggcaccatcctcaa, Antisense – cagctcggccatattcacac. *Trophoblast specific protein alpha (Tpbpa),* Sense – cggaaggctccaacatagaa, Antisense – tcaaattcagggtcatcaacaa. *Mammalian achaete-scute homolog 2 (Mash2)* Sense – TTTTCGAGGACGCAATAAGC, Antisense – cactgctgcaggactcccta. Statistical analysis of real time PCR results were performed as follows: All data points were performed in triplicate. One-way analysis of variance of the results was performed in Microsoft Excel 2007 to determine the presence of significant differences within the data sets. When analysis of variance indicated that a significant difference may be present, a two-sample Student’s t-test was performed to compare experimental data with appropriate controls [77]. Statistical significance was determined at a value of P<0.05 and is represented with an asterisk.

### Plasmid Constructs and Ectopic Expression

The full-length open reading frame of Hif-1α and Hif-2α were PCR amplified from cDNA constructs. Deletional mutants were generated removing the basic domain, Hif-1αΔb (deletion of amino acids 4–27) and Hif-2αΔb (deletion of amino acids 6–24) by high fidelity PCR. All expression constructs were modified to include the 9 amino acid hemagglutinin epitope YPYDVPDYA fused directly to the C-terminus and cloned into the ENTRD-TOPO vector (Invitrogen). The integrity of the constructs was confirmed by DNA sequencing. For expression in cell culture, a derivative of the Piggybac transposon system was employed allowing high efficiency expression. The parental plasmid EBXN containing the minimal Piggybac 5′ and 3′ inverted terminal repeats as well as a CMV enhancer chicken Beta-actin promoter expression cassette was modified to include the SV40 promoter Blasticidin cassette allowing for eukaryotic selection in cell culture. The plasmid was further modified to include the Invitrogen Gateway Rfa cassette allowing for phiC31 mediated recombination. For monitoring transfection efficiency, EMCV IRES upstream of palmitoylated EGFP was inserted, PBX2.2. A control construct containing monomeric EGFP (Karel Svoboda, Addgene Plasmid 18696) was inserted into the parental plasmid PBX2.1.

Transfection of TSC lines was performed with Lipofectamine LTX and PLUS reagent (Invitrogen) in placental fibroblast-free culture. A 2∶1 molar ratio of Piggybac transposase helper plasmid, PB, was combined with the transposon expression construct to mediate integration and high level expression. Selection with Blasticidin 5 mcg/ml was performed to identify stable integrants, which were subsequently passaged on placental fibroblasts.

## References

[1] Simon MC, Keith B (2008) The role of oxygen availability in embryonic development and stem cell function. Nat Rev Mol Cell Biol 9: 285–296. 18285802 10.1038/nrm2354 PMC2876333

[2] Semenza GL (2009) Regulation of oxygen homeostasis by hypoxia-inducible factor 1. Physiology (Bethesda) 24: 97–106. 19364912 10.1152/physiol.00045.2008

[3] Kaelin WG Jr, Ratcliffe PJ (2008) Oxygen sensing by metazoans: the central role of the HIF hydroxylase pathway. Mol Cell 30: 393–402. 18498744 10.1016/j.molcel.2008.04.009

[4] Wang GL, Jiang BH, Rue EA, Semenza GL (1995) Hypoxia-inducible factor 1 is a basic-helix-loop-helix-PAS heterodimer regulated by cellular O2 tension. Proc Natl Acad Sci U S A 92: 5510–5514. 7539918 10.1073/pnas.92.12.5510 PMC41725

[5] Chandel NS, Maltepe E, Goldwasser E, Mathieu CE, Simon MC, et al (1998) Mitochondrial reactive oxygen species trigger hypoxia-induced transcription. Proc Natl Acad Sci U S A 95: 11715–11720. 9751731 10.1073/pnas.95.20.11715 PMC21706

[6] Dunwoodie SL (2009) The role of hypoxia in development of the Mammalian embryo. Dev Cell 17: 755–773. 20059947 10.1016/j.devcel.2009.11.008

[7] Ryan HE, Lo J, Johnson RS (1998) HIF-1 alpha is required for solid tumor formation and embryonic vascularization. Embo J 17: 3005–3015. 9606183 10.1093/emboj/17.11.3005 PMC1170640

[8] Iyer NV, Kotch LE, Agani F, Leung SW, Laughner E, et al (1998) Cellular and developmental control of O2 homeostasis by hypoxia-inducible factor 1 alpha. Genes Dev 12: 149–162. 9436976 10.1101/gad.12.2.149 PMC316445

[9] Maltepe E, Schmidt JV, Baunoch D, Bradfield CA, Simon MC (1997) Abnormal angiogenesis and responses to glucose and oxygen deprivation in mice lacking the protein ARNT. Nature 386: 403–407. 9121557 10.1038/386403a0

[10] Cowden Dahl KD, Fryer BH, Mack FA, Compernolle V, Maltepe E, et al (2005) Hypoxia-inducible factors 1alpha and 2alpha regulate trophoblast differentiation. Mol Cell Biol 25: 10479–10491. 16287860 10.1128/MCB.25.23.10479-10491.2005 PMC1291235

[11] Kozak KR, Abbott B, Hankinson O (1997) ARNT-deficient mice and placental differentiation. Dev Biol 191: 297–305. 9398442 10.1006/dbio.1997.8758

[12] Adelman DM, Gertsenstein M, Nagy A, Simon MC, Maltepe E (2000) Placental cell fates are regulated in vivo by HIF-mediated hypoxia responses. Genes Dev 14: 3191–3203. 11124810 10.1101/gad.853700 PMC317149

[13] Maltepe E, Bakardjiev AI, Fisher SJ (2012) The placenta: transcriptional, epigenetic, and physiological integration during development. J Clin Invest 120: 1016–1025. 10.1172/JCI41211 PMC2846055 20364099

[14] Rossant J, Cross JC (2001) Placental development: lessons from mouse mutants. Nat Rev Genet 2: 538–548. 11433360 10.1038/35080570

[15] Watson ED, Cross JC (2005) Development of structures and transport functions in the mouse placenta. Physiology (Bethesda) 20: 180–193. 15888575 10.1152/physiol.00001.2005

[16] Tanaka S, Kunath T, Hadjantonakis AK, Nagy A, Rossant J (1998) Promotion of trophoblast stem cell proliferation by FGF4. Science 282: 2072–2075. 9851926 10.1126/science.282.5396.2072

[17] Maltepe E, Krampitz GW, Okazaki KM, Red-Horse K, Mak W, et al (2005) Hypoxia-inducible factor-dependent histone deacetylase activity determines stem cell fate in the placenta. Development 132: 3393–3403. 15987772 10.1242/dev.01923

[18] Zheng QA, Chang DC (1991) Reorganization of cytoplasmic structures during cell fusion. J Cell Sci 100 (Pt 3): 431–442. 10.1242/jcs.100.3.431 1808197

[19] Shibukawa Y, Yamazaki N, Kumasawa K, Daimon E, Tajiri M, et al.. (2012) Calponin 3 Regulates Actin Cytoskeleton Rearrangement in Trophoblastic Cell Fusion. Mol Biol Cell. 10.1091/mbc.E10-03-0261 PMC2982094 20861310

[20] Yoshie M, Kashima H, Bessho T, Takeichi M, Isaka K, et al (2008) Expression of stathmin, a microtubule regulatory protein, is associated with the migration and differentiation of cultured early trophoblasts. Hum Reprod 23: 2766–2774. 18718898 10.1093/humrep/den317

[21] Gauster M, Siwetz M, Orendi K, Moser G, Desoye G, et al (2012) Caspases rather than calpains mediate remodelling of the fodrin skeleton during human placental trophoblast fusion. Cell Death Differ 17: 336–345. 10.1038/cdd.2009.133 19798107

[22] Swistowski A, Peng J, Han Y, Swistowska AM, Rao MS, et al (2009) Xeno-free defined conditions for culture of human embryonic stem cells, neural stem cells and dopaminergic neurons derived from them. PLoS One 4: e6233. 19597550 10.1371/journal.pone.0006233 PMC2705186

[23] Swistowski A, Peng J, Liu Q, Mali P, Rao MS, et al (2012) Efficient generation of functional dopaminergic neurons from human induced pluripotent stem cells under defined conditions. Stem Cells 28: 1893–1904. 10.1002/stem.499 PMC2996088 20715183

[24] Hughes CS, Radan L, Betts D, Postovit LM, Lajoie GA (2012) Proteomic analysis of extracellular matrices used in stem cell culture. Proteomics 11: 3983–3991. 10.1002/pmic.201100030 21834137

[25] Damsky CH, Fitzgerald ML, Fisher SJ (1992) Distribution patterns of extracellular matrix components and adhesion receptors are intricately modulated during first trimester cytotrophoblast differentiation along the invasive pathway, in vivo. J Clin Invest 89: 210–222. 1370295 10.1172/JCI115565 PMC442839

[26] Armant DR (2005) Blastocysts don’t go it alone. Extrinsic signals fine-tune the intrinsic developmental program of trophoblast cells. Dev Biol 280: 260–280. 15882572 10.1016/j.ydbio.2005.02.009 PMC2715296

[27] Asanoma K, Kato H, Yamaguchi S, Shin CH, Liu ZP, et al (2007) HOP/NECC1, a novel regulator of mouse trophoblast differentiation. J Biol Chem 282: 24065–24074. 17576768 10.1074/jbc.M701380200

[28] Hynes RO (2009) The extracellular matrix: not just pretty fibrils. Science 326: 1216–1219. 19965464 10.1126/science.1176009 PMC3536535

[29] Hamanaka RB, Chandel NS (2012) Mitochondrial reactive oxygen species regulate cellular signaling and dictate biological outcomes. Trends Biochem Sci 35: 505–513. 10.1016/j.tibs.2010.04.002 PMC2933303 20430626

[30] Page EL, Robitaille GA, Pouyssegur J, Richard DE (2002) Induction of hypoxia-inducible factor-1alpha by transcriptional and translational mechanisms. J Biol Chem 277: 48403–48409. 12379645 10.1074/jbc.M209114200

[31] Richard DE, Berra E, Gothie E, Roux D, Pouyssegur J (1999) p42/p44 mitogen-activated protein kinases phosphorylate hypoxia-inducible factor 1alpha (HIF-1alpha) and enhance the transcriptional activity of HIF-1. J Biol Chem 274: 32631–32637. 10551817 10.1074/jbc.274.46.32631

[32] Emerling BM, Platanias LC, Black E, Nebreda AR, Davis RJ, et al (2005) Mitochondrial reactive oxygen species activation of p38 mitogen-activated protein kinase is required for hypoxia signaling. Mol Cell Biol 25: 4853–4862. 15923604 10.1128/MCB.25.12.4853-4862.2005 PMC1140591

[33] Craig EA, Stevens MV, Vaillancourt RR, Camenisch TD (2008) MAP3Ks as central regulators of cell fate during development. Dev Dyn 237: 3102–3114. 18855897 10.1002/dvdy.21750

[34] Campbell ID, Humphries MJ (2011) Integrin structure, activation, and interactions. Cold Spring Harb Perspect Biol 3. 10.1101/cshperspect.a004994 PMC3039929 21421922

[35] Butcher DT, Alliston T, Weaver VM (2009) A tense situation: forcing tumour progression. Nat Rev Cancer 9: 108–122. 19165226 10.1038/nrc2544 PMC2649117

[36] Scales TM, Parsons M (2011) Spatial and temporal regulation of integrin signalling during cell migration. Curr Opin Cell Biol 23: 562–568. 21696935 10.1016/j.ceb.2011.05.008

[37] Parast MM, Aeder S, Sutherland AE (2001) Trophoblast giant-cell differentiation involves changes in cytoskeleton and cell motility. Dev Biol 230: 43–60. 11161561 10.1006/dbio.2000.0102

[38] Scott RW, Olson MF (2007) LIM kinases: function, regulation and association with human disease. J Mol Med (Berl) 85: 555–568. 17294230 10.1007/s00109-007-0165-6

[39] Minamide LS, Striegl AM, Boyle JA, Meberg PJ, Bamburg JR (2000) Neurodegenerative stimuli induce persistent ADF/cofilin-actin rods that disrupt distal neurite function. Nat Cell Biol 2: 628–636. 10980704 10.1038/35023579

[40] Yusa K, Rad R, Takeda J, Bradley A (2009) Generation of transgene-free induced pluripotent mouse stem cells by the piggyBac transposon. Nat Methods 6: 363–369. 19337237 10.1038/nmeth.1323 PMC2677165

[41] Jiang BH, Rue E, Wang GL, Roe R, Semenza GL (1996) Dimerization, DNA binding, and transactivation properties of hypoxia-inducible factor 1. J Biol Chem 271: 17771–17778. 8663540 10.1074/jbc.271.30.17771

[42] Koshiji M, Kageyama Y, Pete EA, Horikawa I, Barrett JC, et al (2004) HIF-1alpha induces cell cycle arrest by functionally counteracting Myc. Embo J 23: 1949–1956. 15071503 10.1038/sj.emboj.7600196 PMC404317

[43] Gordan JD, Bertout JA, Hu CJ, Diehl JA, Simon MC (2007) HIF-2alpha promotes hypoxic cell proliferation by enhancing c-myc transcriptional activity. Cancer Cell 11: 335–347. 17418410 10.1016/j.ccr.2007.02.006 PMC3145406

[44] Scott RW, Hooper S, Crighton D, Li A, Konig I, et al (2012) LIM kinases are required for invasive path generation by tumor and tumor-associated stromal cells. J Cell Biol 191: 169–185. 10.1083/jcb.201002041 PMC2953444 20876278

[45] Ross-Macdonald P, de Silva H, Guo Q, Xiao H, Hung CY, et al (2008) Identification of a nonkinase target mediating cytotoxicity of novel kinase inhibitors. Mol Cancer Ther 7: 3490–3498. 19001433 10.1158/1535-7163.MCT-08-0826

[46] Guilak F, Cohen DM, Estes BT, Gimble JM, Liedtke W, et al (2009) Control of stem cell fate by physical interactions with the extracellular matrix. Cell Stem Cell 5: 17–26. 19570510 10.1016/j.stem.2009.06.016 PMC2768283

[47] Kim SH, Turnbull J, Guimond S (2012) Extracellular matrix and cell signalling: the dynamic cooperation of integrin, proteoglycan and growth factor receptor. J Endocrinol 209: 139–151. 10.1530/JOE-10-0377 21307119

[48] Votteler M, Kluger PJ, Walles H, Schenke-Layland K (2012) Stem cell microenvironments–unveiling the secret of how stem cell fate is defined. Macromol Biosci 10: 1302–1315. 10.1002/mabi.201000102 20715131

[49] Eshghi S, Vogelezang MG, Hynes RO, Griffith LG, Lodish HF (2007) Alpha4beta1 integrin and erythropoietin mediate temporally distinct steps in erythropoiesis: integrins in red cell development. J Cell Biol 177: 871–880. 17548514 10.1083/jcb.200702080 PMC2064286

[50] Skuli N, Monferran S, Delmas C, Favre G, Bonnet J, et al (2009) Alphavbeta3/alphavbeta5 integrins-FAK-RhoB: a novel pathway for hypoxia regulation in glioblastoma. Cancer Res 69: 3308–3316. 19351861 10.1158/0008-5472.CAN-08-2158

[51] Cowden Dahl KD, Robertson SE, Weaver VM, Simon MC (2005) Hypoxia-inducible factor regulates alphavbeta3 integrin cell surface expression. Mol Biol Cell 16: 1901–1912. 15689487 10.1091/mbc.E04-12-1082 PMC1073670

[52] Wang J, Armant DR (2002) Integrin-mediated adhesion and signaling during blastocyst implantation. Cells, tissues, organs 172: 190–201. 12476048 10.1159/000066970

[53] Sutherland A (2003) Mechanisms of implantation in the mouse: differentiation and functional importance of trophoblast giant cell behavior. Dev Biol 258: 241–251. 12798285 10.1016/s0012-1606(03)00130-1

[54] Rosario GX, Konno T, Soares MJ (2008) Maternal hypoxia activates endovascular trophoblast cell invasion. Dev Biol 314: 362–375. 18199431 10.1016/j.ydbio.2007.12.007 PMC2266816

[55] Ramos JW (2008) The regulation of extracellular signal-regulated kinase (ERK) in mammalian cells. Int J Biochem Cell Biol 40: 2707–2719. 18562239 10.1016/j.biocel.2008.04.009

[56] Hsu JC, Perrimon N (1994) A temperature-sensitive MEK mutation demonstrates the conservation of the signaling pathways activated by receptor tyrosine kinases. Genes Dev 8: 2176–2187. 7958887 10.1101/gad.8.18.2176

[57] Wu Y, Han M, Guan KL (1995) MEK-2, a Caenorhabditis elegans MAP kinase kinase, functions in Ras-mediated vulval induction and other developmental events. Genes Dev 9: 742–755. 7729690 10.1101/gad.9.6.742

[58] Umbhauer M, Marshall CJ, Mason CS, Old RW, Smith JC (1995) Mesoderm induction in Xenopus caused by activation of MAP kinase. Nature 376: 58–62. 7541116 10.1038/376058a0

[59] Kornfeld K, Guan KL, Horvitz HR (1995) The Caenorhabditis elegans gene mek-2 is required for vulval induction and encodes a protein similar to the protein kinase MEK. Genes Dev 9: 756–768. 7729691 10.1101/gad.9.6.756

[60] Hamanaka RB, Chandel NS (2009) Mitochondrial reactive oxygen species regulate hypoxic signaling. Curr Opin Cell Biol 21: 894–899. 19781926 10.1016/j.ceb.2009.08.005 PMC2787901

[61] Harburger DS, Calderwood DA (2009) Integrin signalling at a glance. J Cell Sci 122: 159–163. 19118207 10.1242/jcs.018093 PMC2714413

[62] Bissonauth V, Roy S, Gravel M, Guillemette S, Charron J (2006) Requirement for Map2k1 (Mek1) in extra-embryonic ectoderm during placentogenesis. Development 133: 3429–3440. 16887817 10.1242/dev.02526

[63] Saba-El-Leil MK, Vella FD, Vernay B, Voisin L, Chen L, et al (2003) An essential function of the mitogen-activated protein kinase Erk2 in mouse trophoblast development. EMBO Rep 4: 964–968. 14502223 10.1038/sj.embor.embor939 PMC1326397

[64] Hatano N, Mori Y, Oh-hora M, Kosugi A, Fujikawa T, et al (2003) Essential role for ERK2 mitogen-activated protein kinase in placental development. Genes Cells 8: 847–856. 14622137 10.1046/j.1365-2443.2003.00680.x

[65] Giroux S, Tremblay M, Bernard D, Cardin-Girard JF, Aubry S, et al (1999) Embryonic death of Mek1-deficient mice reveals a role for this kinase in angiogenesis in the labyrinthine region of the placenta. Curr Biol 9: 369–372. 10209122 10.1016/s0960-9822(99)80164-x

[66] Nadeau V, Guillemette S, Belanger LF, Jacob O, Roy S, et al (2009) Map2k1 and Map2k2 genes contribute to the normal development of syncytiotrophoblasts during placentation. Development 136: 1363–1374. 19304888 10.1242/dev.031872

[67] Galabova-Kovacs G, Matzen D, Piazzolla D, Meissl K, Plyushch T, et al (2006) Essential role of B-Raf in ERK activation during extraembryonic development. Proc Natl Acad Sci U S A 103: 1325–1330. 16432225 10.1073/pnas.0507399103 PMC1360532

[68] Dubois NC, Adolphe C, Ehninger A, Wang RA, Robertson EJ, et al (2008) Placental rescue reveals a sole requirement for c-Myc in embryonic erythroblast survival and hematopoietic stem cell function. Development 135: 2455–2465. 18550708 10.1242/dev.022707

[69] Fukuda R, Hirota K, Fan F, Jung YD, Ellis LM, et al (2002) Insulin-like growth factor 1 induces hypoxia-inducible factor 1-mediated vascular endothelial growth factor expression, which is dependent on MAP kinase and phosphatidylinositol 3-kinase signaling in colon cancer cells. J Biol Chem 277: 38205–38211. 12149254 10.1074/jbc.M203781200

[70] Lee SH, Che X, Jeong JH, Choi JY, Lee YJ, et al (2012) Runx2 protein stabilizes hypoxia-inducible factor-1alpha through competition with von Hippel-Lindau protein (pVHL) and stimulates angiogenesis in growth plate hypertrophic chondrocytes. J Biol Chem 287: 14760–14771. 22351759 10.1074/jbc.M112.340232 PMC3340285

[71] Escuin D, Kline ER, Giannakakou P (2005) Both microtubule-stabilizing and microtubule-destabilizing drugs inhibit hypoxia-inducible factor-1alpha accumulation and activity by disrupting microtubule function. Cancer Res 65: 9021–9028. 16204076 10.1158/0008-5472.CAN-04-4095 PMC6623969

[72] Mabjeesh NJ, Escuin D, LaVallee TM, Pribluda VS, Swartz GM, et al (2003) 2ME2 inhibits tumor growth and angiogenesis by disrupting microtubules and dysregulating HIF. Cancer Cell 3: 363–375. 12726862 10.1016/s1535-6108(03)00077-1

[73] Burton GJ (2009) Oxygen, the Janus gas; its effects on human placental development and function. J Anat 215: 27–35. 19175804 10.1111/j.1469-7580.2008.00978.x PMC2714636

[74] Pringle KG, Kind KL, Sferruzzi-Perri AN, Thompson JG, Roberts CT (2009) Beyond oxygen: complex regulation and activity of hypoxia inducible factors in pregnancy. Hum Reprod Update. 10.1093/humupd/dmp046 PMC2880912 19926662

[75] Zhang L, Luo J, Wan P, Wu J, Laski F, et al (2012) Regulation of cofilin phosphorylation and asymmetry in collective cell migration during morphogenesis. Development 138: 455–464. 10.1242/dev.046870 21205790

[76] Karthikeyan VJ, Lane DA, Beevers DG, Lip GY, Blann AD (2012) Matrix metalloproteinases and their tissue inhibitors in hypertension-related pregnancy complications. J Hum Hypertens. 10.1038/jhh.2012.8 22418748

[77] Rieu I, Powers SJ (2009) Real-time quantitative RT-PCR: design, calculations, and statistics. Plant Cell 21: 1031–1033. 19395682 10.1105/tpc.109.066001 PMC2685626

